# Osteosarcopenia: key molecular mechanisms and translational perspectives

**DOI:** 10.3389/fphys.2025.1723522

**Published:** 2026-01-09

**Authors:** Yuan Pu, Yirong Teng, Yinghua Li, Yunchun Zhou, Ming Gao, Zilin Yan, Zhaowei Teng

**Affiliations:** 1 Department of General Medicine, The Sixth Affiliated Hospital of Kunming Medical University, Yuxi, China; 2 Department of Center Laboratory, The Second Affiliated Hospital of Kunming Medical University, Kunming, China

**Keywords:** osteosarcopenia, osteoporosis, sarcopenia, molecular mechanisms, clinical transformation

## Abstract

The coexistence of osteoporosis and sarcopenia is recognized as a syndrome known as osteosarcopenia. As an aging-related disease, research into the molecular mechanisms of osteosarcopenia has gradually shifted from the study of single-organ pathology to the elucidation of multidimensional interactions. This review aims to construct a hierarchical framework of “intracellular - intercellular - systemic” to systematically elaborate on the pathogenesis of osteosarcopenia. Based on this foundation, it explores frontier interventions and their prospects for clinical transformation, including bone-targeting F6-(DSS)6-exo nanoparticles, miR-495, natural active compounds (resveratrol, nuciferine), *Clostridium* butyricum, and bimagrumab. Future research should focus on analyzing the microenvironment of the musculoskeletal interface, utilizing deep learning CT analysis for early risk identification, and exploring the application of biomaterials in osteomuscular regeneration. This review aims to provide a reference for the field of mechanism research in osteosarcopenia and offer new insights for its precision prevention and treatment.

## 1 Introduction

The skeletal and muscular systems, fundamental pillars of human locomotion, do not function in isolation; rather, they represent intricate, interconnected tissues exhibiting profound synergistic interactions. Bone and muscle tissues exhibit remarkable co-evolutionary changes. This phenomenon is observed throughout individual development, growth, and aging. It also extends to the progression of numerous pathologies (Gielen et al., 2023). As the aging process unfolds, both skeletal and muscular morphology and function progressively decline. This manifests as a relentless reduction in bone mineral density (BMD) and deterioration of bone tissue microarchitecture, culminating in osteoporosis, a systemic skeletal disorder characterized by heightened bone fragility and a significantly elevated fracture risk (Ensrud and Crandall, 2024). Concurrently, skeletal muscle mass, strength, and overall function experience a parallel decline, progressing into sarcopenia. This progressive, generalized skeletal muscle disorder is strongly associated with an increased risk of falls, functional impairment, and mortality (Cruz-Jentoft and Sayer, 2019). The global population is rapidly aging. This demographic shift has led to a dramatic surge in the prevalence of both osteoporosis and sarcopenia. As a result, these conditions now pose critical public health challenges (Gao et al., 2021; Salari et al., 2021).

Our comprehension of the intricate “bone-muscle crosstalk” mechanisms governing the interplay between skeletal and muscular tissues is continuously advancing. This bidirectional interaction implies that diminished bone quality can directly or indirectly precipitate a reduction in muscle mass and function. Conversely, a decrease in muscle mass has been shown to accelerate the loss of bone mineral density and trabecular architecture (Yu et al., 2023). Bidirectional mendelian randomization studies, leveraging genetic variants as instrumental variables to effectively mitigate confounding, have provided compelling evidence. These investigations suggest that osteoporosis may represent a significant risk factor for sarcopenia-related phenotypes, including muscle mass and strength. Furthermore, muscle strength itself can influence BMD via gene-environment interactions. Collectively, these findings underscore a substantial causal relationship between osteoporosis and sarcopenia, offering crucial genetic epidemiological support (Ma et al., 2022). Further research indicates a bidirectional relationship. Individuals with severe osteoporosis are more susceptible to appendicular lean mass (ALM) loss. Conversely, significant ALM deficits can contribute to reduced lumbar spine BMD (LS BMD) (Liu et al., 2022).

Recognizing the profound functional interdependence of bone and muscle, the novel geriatric syndrome of osteosarcopenia has emerged. Its defining characteristic is the concomitant presence of reduced BMD (encompassing both osteopenia and osteoporosis) alongside diminished skeletal muscle mass and/or function (sarcopenia) (Kirk et al., 2020b). Osteosarcopenia exhibits a high prevalence among the elderly population and substantially elevates the risk of adverse clinical outcomes, consequently imposing a considerable burden on affected individuals, their families, and healthcare systems. Consequently, a comprehensive understanding of osteosarcopenia’s pathogenesis is paramount for the development of effective preventive and therapeutic strategies, ultimately driving its clinical transformation.

## 2 Molecular interaction mechanisms

**FIGURE 1:**
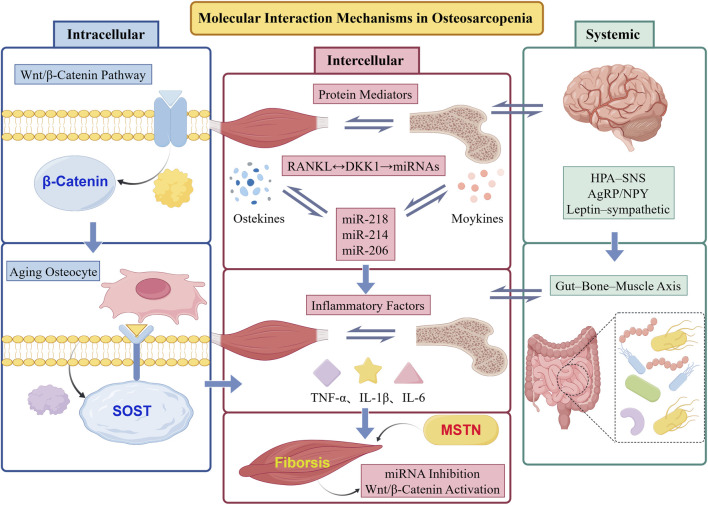
Molecular mechanisms of bone-muscle interaction. The figure created by Figdraw (accessed on 25 December 2025).

The development and progression of osteosarcopenia arise from dysregulation within multilevel molecular networks encompassing intracellular signaling, intercellular communication, and integrated neuro–endocrine–metabolic feedback systems. Understanding the cross-regulation among these molecular hierarchies is fundamental to elucidating the pathophysiological basis and identifying potential therapeutic targets within the bone–muscle unit (Figure 1).

### 2.1 Intracellular autonomous regulation: spatiotemporal activation and signal integration of the Wnt/β-catenin pathway

The Wnt/β-catenin signaling pathway represents one of the central molecular mechanisms maintaining bone–muscle unit homeostasis, characterized by evident context-dependent regulation and cell-type specificity. In osteoblasts, Wnt signaling induces β-catenin nuclear translocation, activating the transcription of osteogenic genes such as Runx2 and Osterix (Hu et al., 2024), while counterbalancing the receptor activator of nuclear factor-κB ligand (RANKL)/receptor activator of nuclear factor-κB (RANK) axis to preserve bone remodeling equilibrium (Noh et al., 2020). During aging, Wnt signaling displays compartment-specific heterogeneity: osteocytes exhibit reduced receptor sensitivity and diminished transcriptional activity, whereas in myogenic progenitors, chronic low-grade inflammation drives excessive Wnt activation, leading to fibrosis and impaired regeneration (Malvandi et al., 2025b). Notably, targeted activation of Wnt signaling in bone marrow mesenchymal stem cells (BMSCs) promotes osteogenic differentiation and bone repair (Matsushita et al., 2020). Osteocytes also secrete Wnt3a via paracrine pathways (e.g., gap junctions) to activate β-catenin signaling in neighboring cells (Sukarawan et al., 2023; Lin et al., 2024). However, most evidence derives from animal aging models and *in vitro* systems, and systemic overactivation of Wnt signaling may induce metabolic uncoupling or heterotopic ossification, highlighting the risk of maladaptive remodeling in the aged bone–muscle system. Sclerostin (SOST), an osteocyte-derived negative regulator, serves as a key inhibitory node of Wnt/β-catenin signaling. Upregulation of SOST suppresses osteogenic signaling and delays bone repair, whereas SOST inhibition enhances osteoblastic activity and improves muscle metabolism (Kirk et al., 2020a; Sheng et al., 2023).

### 2.2 Intercellular communication layer

Bone and muscle are not isolated entities; rather, they engage in complex intercellular communication to exchange information and cooperatively respond to physiological and pathological signals. This intricate regulatory mechanism provides crucial insights into the pathogenesis of osteosarcopenia. Intercellular communication occurs through various modalities, and the following sections will introduce several mediators critically involved in osteosarcopenia.

#### 2.2.1 Bidirectional regulation by protein mediators

Myokines and osteokines directly influence tissue phenotypes via reciprocal modulation. Muscle-derived factors (e.g., myokines secreted by C2C12 myotubes) can activate the osteocytic Wnt/β-catenin pathway, synergizing with Wnt3a signaling to promote osteogenesis (Lara-Castillo et al., 2023). Clinical evidence indicates that increased muscle mass does not necessarily translate into functional improvement, suggesting a potential functional decoupling between muscle morphology and performance (Faraldi et al., 2025). Myostatin (MSTN/GDF8), a member of the TGF-β superfamily, acts as a critical negative regulator within the bone–muscle unit. It inhibits myogenesis via the Smad2/3 pathway while upregulating RANKL expression to enhance osteoclastogenesis (Saad, 2020; Cui et al., 2023). MSTN knockout mice exhibit muscle fiber hypertrophy and increased contractile strength (Degens et al., 2025); however, prolonged MSTN inhibition may lead to bone loss and abnormal muscle architecture (Faraldi et al., 2025), reflecting its dose-dependent and systemically coupled characteristics. At the molecular level, MSTN suppresses microRNA-218 expression in osteocytic exosomes, indirectly increasing SOST and DKK1 levels, thereby forming a negative feedback loop regulating Wnt signaling (Kirk et al., 2020a). Moreover, mechanical loading–induced SOST upregulation can restrict muscle anabolism, underscoring SOST’s dual role as both an osteogenic inhibitor and a modulator of muscle metabolism (Yu et al., 2023).

#### 2.2.2 miRNAs: a novel perspective in bone-muscle metabolic regulation

MicroRNAs (miRNAs) function as pivotal mediators in bone–muscle communication, acting both as signaling effectors and circulating biomarkers (Malvandi et al., 2025a). Osteocyte-derived exosomal miR-218 can be internalized by osteoblasts, suppressing bone formation (Kirk et al., 2020a); similarly, miR-214-3p inhibits osteogenesis in BMSCs via the LINC01133/CTNNB1 axis (Guo et al., 2023; Chen et al., 2024; Tang et al., 2024), while osteoclast-derived miR-23a-5p downregulates Runx2 and activates the YAP1–MT1DP axis to repress osteogenic differentiation (Yang et al., 2020). Conversely, muscle-derived miR-206 and miR-486 upregulate osteogenic genes and enhance bone formation (Malvandi et al., 2025a). These miRNAs constitute a molecular bridge linking bone and muscle metabolism, intersecting with both the Wnt/β-catenin and MSTN pathways to form an integrated regulatory hub. Meta-analyses reveal significant correlations between circulating miRNAs (e.g., miR-21, miR-23a, miR-24, miR-27a) and BMD (Nóbrega et al., 2020). Multi-omics studies highlight the miR-21/23a, miR-133/206, and miR-214 networks as key regulators of aging-related bone–muscle homeostasis (Malvandi et al., 2025a). Nonetheless, interstudy variability (e.g., sample processing and normalization methods) and uncertainty regarding tissue origin remain major barriers to their clinical translation as biomarkers.

#### 2.2.3 Inflammatory factors: regulating bone-muscle balance

Chronic low-grade inflammation disrupts bone–muscle homeostasis. Proinflammatory cytokines such as TNF-α, IL-1β, and IL-6 activate the NF-κB pathway, promoting osteoclastogenesis and inhibiting osteogenesis, thereby contributing to osteoporosis (Jimenez-Gutierrez et al., 2022). Concurrently, they enhance the ubiquitin–proteasome and autophagy pathways in muscle, accelerating protein degradation and sarcopenic atrophy (Silva, 2024). TNF-α exerts dual effects—facilitating acute repair but inhibiting myogenesis and inducing atrophy when chronically elevated—whereas IL-15 maintains muscle mass by upregulating myosin heavy chain expression and suppressing inflammation (Alcalde-Estévez et al., 2025). The antagonism between TNF-α and IL-15 underscores the bidirectional nature of inflammatory signaling and its central role in bone–muscle aging.

### 2.3 Systemic regulation layer

Homeostasis within the bone–muscle unit is orchestrated by the neural, endocrine, and metabolic systems through multilevel integration. The hypothalamic–pituitary–sympathetic (HPA–SNS) axis serves as a central regulator of energy balance and bone–muscle metabolism. AgRP/NPY neurons modulate bone mass, with neuronal ablation leading to increased bone density (Enriquez et al., 2021); leptin–sympathetic signaling via β2-adrenergic receptors upregulates RANKL expression to couple energy expenditure with bone resorption (Shi and Chen, 2024). Chronic stress or aging causes hyperactivation of this axis, leading to concurrent muscle wasting and bone loss, a hallmark of systemic maladaptive signaling. Moreover, muscle-derived myokines can feedback to the central nervous system, modulating neuroinflammation and energy homeostasis, thereby establishing a muscle–brain bidirectional loop (Rai and Demontis, 2022).

Emerging evidence supports the concept of a gut–bone–muscle axis, wherein the gut microbiota regulates skeletal and muscular metabolism via short-chain fatty acids such as butyrate (Li et al., 2021; Mancin et al., 2023). Butyrate enhances protein synthesis, inhibits muscle proteolysis, and improves bone density (Chen et al., 2025), whereas microbial dysbiosis triggers chronic inflammation that perturbs the bone marrow immune microenvironment and impairs bone formation and muscle regeneration (Li et al., 2024). The abundance of anabolic microbial species correlates with ALM and BMD, suggesting that the gut microbiome represents a key systemic regulatory node in bone–muscle homeostasis (Grahnemo et al., 2023).

## 3 Clinical diagnosis and treatment status

### 3.1 Current status of clinical diagnosis

Currently, the Duque team has proposed a clinical diagnosis for osteosarcopenia (Kirk et al., 2020b), organically integrating the consensus of the European working group on sarcopenia in older people (EWGSOP2) with the WHO diagnostic criteria for osteoporosis, combined with dual-energy X-ray absorptiometry (DXA) for a “one-stop” confirmed diagnosis. The specific procedure is as follows: First, screening for impaired muscle function is conducted using the SARC-F questionnaire and handgrip strength measurement; subsequently, DXA is used to measure the ALM index (ALM/height^2^). Sarcopenia is confirmed when the value falls below specific cutoff points (e.g., EWGSOP2 criteria: men <7.0 kg/m^2^, women <5.5 kg/m^2^). Synchronously, BMD is measured; osteoporosis is confirmed if the T-score is ≤−2.5 or if there is a history of fragility fractures. When a patient meets the dual criteria for both sarcopenia and osteoporosis, they are defined as having osteosarcopenia.

Evaluation of existing models reveals that the DXA-based assessment pathway is the preferred screening tool as it acquires bone mineral content and soft tissue composition data simultaneously with low radiation dose and relatively low cost. However, the limitation of this model is that DXA only provides two-dimensional muscle mass (quantity) data and cannot provide precise anatomical details of muscle cross-sectional area, like CT or MRI, nor can it quantify myosteatosis, a key indicator of muscle quality. Additionally, DXA soft tissue measurements are susceptible to the body’s hydration status, leading to certain measurement errors.

The lack of a unified global “gold standard” for diagnosing osteosarcopenia stems not merely from a paucity of data, but from the heterogeneity of basic definitions and ethnic dimorphism. Since sarcopenia itself has multiple international consensuses, such as EWGSOP2 and the Asian working group for sarcopenia (AWGS), differences in diagnostic cutoffs across these standards directly lead to inconsistencies in osteosarcopenia diagnosis. Simultaneously, there is a lack of biomarkers (such as specific miRNA combinations or osteomyokines) capable of sensitively reflecting synchronous bone-muscle loss prior to radiological changes. Therefore, current clinical practice tends to use operational definitions adapted to specific population characteristics rather than a single absolute standard.

### 3.2 Current status of clinical treatment

Currently, treatment approaches for osteosarcopenia are categorized into two main types: non-pharmacological interventions and pharmacological treatments.

#### 3.2.1 Non-pharmacological interventions

Evidence from multiple randomized controlled trials (RCTs) in humans has demonstrated that progressive resistance training effectively stimulates osteoblast activity and muscle protein synthesis, thereby improving bone microarchitecture, muscle mass, strength, and functional performance in individuals with osteosarcopenia (Kirk et al., 2020b). Moreover, data from systematic reviews and observational human studies underscore the critical role of adequate nutritional support. A daily intake of 1.0–1.2 g/kg body weight of high-quality protein (e.g., whey protein) is recommended, along with supplementation of n-3 polyunsaturated fatty acids, vitamin C, and β-carotene. These nutrients have been consistently shown to enhance muscle mass and functional capacity (Cruz-Jentoft and Sayer, 2019; van Dronkelaar et al., 2023). In addition, several clinical trials have confirmed that combined calcium and vitamin D supplementation can increase BMD, improve muscle function, and reduce the incidence of fractures (Fagundes Belchior et al., 2020).

#### 3.2.2 Pharmacological treatment

To date, the U.S. Food and Drug Administration (FDA) has not approved any pharmacological agents specifically indicated for the treatment of osteosarcopenia. Nevertheless, a number of promising compounds are currently under clinical investigation. Given the frequent coexistence of osteoporosis in patients with osteosarcopenia, the management of osteoporosis remains a cornerstone for improving musculoskeletal outcomes. Commonly prescribed anti-resorptive agents, such as bisphosphonates (e.g., alendronate, zoledronic acid) and denosumab, have been shown in clinical trials to significantly increase bone mineral density through inhibition of osteoclastic bone resorption (LeBoff et al., 2022). Denosumab exerts its potent anti-resorptive effect by blocking the RANKL–RANK signaling pathway, thereby markedly reducing bone turnover (Reid and Billington, 2022). In the realm of osteoanabolic therapy, teriparatide and romosozumab have demonstrated substantial efficacy in enhancing BMD and reducing fracture risk, particularly in patients at high or very high fracture risk. A recent systematic review and meta-analysis revealed that teriparatide yields a significantly greater increase in lumbar spine BMD among postmenopausal osteoporotic women compared to bisphosphonates, with a lower incidence of systemic adverse events (Yang et al., 2024). Romosozumab, both *in vitro* and in animal models, has been shown to inhibit sclerostin, thereby activating the Wnt signaling pathway to promote bone formation and suppress bone resorption. Clinical studies further corroborate its ability to markedly increase BMD and reduce fracture incidence, making it suitable for patients with very high fracture risk and low cardiovascular risk profiles (Lim, 2022). Selective estrogen receptor modulators (SERMs), such as raloxifene, have been validated in randomized clinical trials to improve BMD and reduce fracture risk in postmenopausal women (Rolland et al., 2023). Exogenous calcitonin, particularly salmon calcitonin, has been shown in short-term clinical studies to suppress bone resorption and alleviate bone pain, with a mild positive effect on BMD. However, its efficacy in preventing non-vertebral or overall fractures remains limited compared with bisphosphonates and other first-line anti-resorptive therapies (Song et al., 2022). Regarding sarcopenia, there are currently no FDA-approved pharmacological agents with disease-modifying potential; nonetheless, several drugs have shown promising preliminary results in human studies. Testosterone replacement therapy (TRT) has been demonstrated to significantly enhance muscle mass, strength, and BMD in hypogonadal men (Barbonetti et al., 2020). Estrogens and their analogues (e.g., raloxifene, tibolone) have been reported in observational studies of postmenopausal women to increase lean body mass by modulating muscle metabolism (Rolland et al., 2023). Given the specific indications and potential adverse effects associated with these agents, careful clinical judgment and individualized treatment selection are essential in optimizing therapeutic outcomes for patients with osteosarcopenia.

### 3.3 Existing treatment limitations

Current pharmacological interventions are predominantly limited to single-tissue targeting; the absence of a single molecular agent capable of concurrently stimulating osteogenesis and muscle protein synthesis has prevented the realization of genuine “bone-muscle synchronous treatment”. Many interventions (including nutrition and drugs) can significantly increase muscle mass, but the patient’s grip strength, gait speed, or fall risk do not improve synchronously. This structure-function uncoupling is a major difficulty in current clinical transformation, suggesting that the importance of neural control or muscle quality (e.g., fiber type conversion) has been underestimated.

## 4 Clinical translation pathways: bridging basic research to clinical application

### 4.1 Strategies targeting bone repair

Addressing the off-target effects of systemic administration, utilizing nanocarriers for precise drug delivery is a frontier direction in regenerative medicine. Research shows that genetically engineered bone-targeting F6-(DSS)6-exo nanoparticles can precisely deliver curcumin (a ferroptosis inhibitor) to bone tissue and BMSCs, effectively promoting bone repair (Wang et al., 2025). Another strategy utilizes a composite hydrogel (GelMA/SAMA/β-TCP) loaded with Exo-Yoda1 (a Piezo1 agonist), which promotes osteoinduction by activating osteogenic signaling pathways (such as ERK), offering a new strategy for bone defect repair (He et al., 2024). While these strategies show potential for precise regulation, they are primarily based on *in vitro* and rodent *in vivo* models. Clinical translation faces challenges: Wnt signaling carries a risk of chronic overactivation in aging tissues (Malvandi et al., 2025b). If nanocarrier delivery of agonists cannot achieve nanosecond-scale “pulsatile” administration, it may trigger fibrosis or cytokine storms in elderly patients rather than the intended regeneration. Therefore, future carrier designs must incorporate “temporal” controlled-release mechanisms.

### 4.2 Anti-myostatin strategies

As a core negative regulator of bone and muscle, MSTN is the target with the highest maturity in clinical transformation. Bimagrumab (ActRIIB monoclonal antibody) is the investigational drug with the highest level of evidence currently. Multiple clinical trials indicate it can safely and effectively increase lean body mass and reduce body fat in elderly and obese populations (Rooks et al., 2020). However, it is noteworthy that previous drugs targeting the MSTN pathway were often terminated due to side effects (such as epistaxis, telangiectasia), and the long-term safety of bimagrumab still needs verification in large-scale phase III trials. To overcome the high cost and side effects of antibody drugs, natural products and nucleic acid drugs have become new directions. Nuciferine has shown the ability to block the MSTN/ActRIIB interaction and downregulate MuRF-1/MAFbx in animal models, ultimately promoting muscle regeneration (Zheng et al., 2024); meanwhile, miR-495 significantly enhanced muscle mass and muscle fiber cross-sectional area (CSA) in cell models by targeting the MyoD/MSTN axis (Zhang et al., 2025). Nucleic acid drugs targeting miR-206/214 have improved bone–muscle metabolism in animals but are still limited by tissue-specific delivery and expression variability (Malvandi et al., 2025a). Although Bimagrumab is effective in increasing muscle mass, the inconsistency in functional improvement and potential compromise of bone quality suggest that therapeutic endpoints must be redefined from the perspective of “bone–muscle crosstalk” (Faraldi et al., 2025).

### 4.3 Metabolic and microbial intervention strategies

These strategies aim to indirectly maintain bone-muscle homeostasis by improving the systemic metabolic environment, characterized by pleiotropic effects. Cellular studies show that resveratrol can promote mitochondrial biogenesis by activating the AMPK/PGC-1α axis and induce the conversion of muscle fibers from easily fatigued fast-twitch fibers to fatigue-resistant slow-twitch fibers (Zhang et al., 2023). However, this remains at the level of *in vitro* mechanism exploration, lacking translational evidence in complex *in vivo* environments. The regulatory role of the gut-musculoskeletal axis has been preliminarily validated in animal models. For example, *Clostridium* butyricum combined with vitamin D3 supplementation significantly increased the abundance of major short-chain fatty acid-producing bacteria, *Alistipes* and *Bacteroidetes*, improving skeletal strength in broiler models (Cao et al., 2024). Additionally, folic acid reduces bone loss via the TGR5/AMPK pathway, and its supplementation can prevent bone loss caused by high body fat, though this research is also largely limited to mouse models (Zhang et al., 2024). The advantages of such interventions lie in high safety and good compliance. However, the evidence is primarily derived from *in vitro* cells or non-primate animals (even avians), presenting massive species differences. For instance, the bioavailability of phytochemicals like resveratrol is extremely low in humans, and the complexity of the human gut microbiota far exceeds that of experimental animals, resulting in simple strain supplementation often failing to replicate the significant efficacy seen in animal experiments clinically (Table 1).

**TABLE 1:** Summaryoverview of clinical translation pathways (not a source of novel data).

Clinical translation pathways	Intervention strategies	Mechanisms	Research stage	References
Strategies targeting bone repair	Curcumin-loaded bone-targeting F6-(DSS)6-exo nanoparticles	Deliver ferroptosis inhibitor to bone tissue and BMSCs to promote bone repair	Preclinical (*in vitro*and animal)	Wang et al. (2025)
	Exo-Yoda1-loaded GelMA/SAMA/β-TCP hydrogels	Activate osteogenic signaling pathways to enhance BMSCs osteogenic differentiation	Preclinical (*in vitro*and animal)	He et al. (2024)
Anti-myostatin strategies	MiR-495 overexpression	Target MyoD and inactivate MSTN/TGF-β/Smad3 signaling pathway to enhance muscle regeneration	Preclinical (*in vitro*and animal)	Zhang et al. (2025)
	Monoclonal antibody Bimagrumab (MSTN inhibitor)	Safely and effectively increase thigh muscle volume, lean body mass, and reduce body fat in older adults and obese individuals	Early clinical (human)	Rooks et al. (2020)
	Nuciferine (alkaloid from *nelumbo nucifera*)	Interfere with MSTN-ActRIIB interaction, downregulate MuRF-1 and MAFbx/Atrogin-1 to promote muscle regeneration	Preclinical (*in vitro*and animal)	Zheng et al. (2024)
Strategies targeting muscle fiber type transformation	Resveratrol	Activate AMPK/PGC-1α signaling pathway to promote mitochondrial biogenesis and induce fast-twitch to slow-twitch muscle fiber type shift	Preclinical (*in vitro*)	Zhang et al. (2023)
Microbiome intervention	Co-supplementation of *Clostridium*butyricum and 25-hydroxyvitamin D3 in broiler feed	Increase abundance of short-chain fatty acid-producing bacteria, regulate mediators via gut-brain axis to increase tibia length, BMD, and bone strength	Preclinical (animal)	Cao et al. (2024)
Nutritional intervention strategies	Folic acid supplementation	Promote LCA and TGR5 expression, increase AMPK phosphorylation, reduce NF-κB and ERK phosphorylation to mitigate bone loss	Preclinical (animal)	Zhang et al. (2024)

BMSCs, bone marrow mesenchymal stem cells; GelMA, gelatin methacryloyl; SAMA, sodium alginate methacrylate; β-TCP, beta-tricalcium phosphate; ERK, extracellular signal-regulated kinase; miR-495, microRNA-495; MyoD, myogenic differentiation 1; MSTN, myostatin; TGF-β, rransforming growth factor-beta; ActRIIB, activin receptor type IIB; MuRF-1, muscle ring finger protein-1; MAFbx, muscle atrophy F-box; AMPK, adenosine monophosphate-activated protein kinase; PGC-1α, peroxisome proliferator-A\activated receptor-γ coactivator-1α; NF-κB, nuclear factor-kappa B; BMD, bone mineral density; LCA, lithocholic acid; TGR5, G-protein-coupled bile acid receptor 1.

Looking at the above strategies, the transformation from basic research to clinical application still faces severe challenges. First, existing evidence relies heavily on chemically induced acute bone-muscle injury animal models, which cannot fully simulate the chronic, age-related, and multi-morbidity characteristics of human osteosarcopenia, limiting the extrapolation of efficacy. Second, osteosarcopenia is part of systemic aging; strategies targeting muscle alone (e.g., MSTN antibodies) or bone alone (e.g., bone-targeted exosomes) may ignore bone-muscle interactions and the influence of the systemic endocrine environment. Furthermore, the pharmacokinetic gap—especially for natural products (e.g., curcumin, resveratrol, nuciferine)—where high concentrations effective in cell experiments are difficult to achieve via oral administration in humans, makes the development of high-bioavailability delivery systems (such as the aforementioned F6-(DSS)6-exo) a critical path to resolving this contradiction.

## 5 Future challenges and breakthrough directions: a new journey in the prevention and treatment of osteosarcopenia

The future landscape of osteosarcopenia prevention and treatment is poised for significant advancements, driven by innovative technologies and multidisciplinary approaches. One major breakthrough lies in advanced diagnostic and research tools. The Visium cytAssist spatial transcriptomics platform has, for the first time, achieved a spatially complete characterization of gene expression changes during fracture healing, particularly in orthopedic models of impaired fracture healing (Jiang et al., 2024). By precisely localizing gene expression, Visium spatial transcriptomics offers deep insights into the bone-muscle interface microenvironment, providing a powerful new research tool for the orthopedic field. Nevertheless, its clinical translation faces many practical hurdles. A primary challenge is the inherent invasiveness required for clinical sample procurement. Another significant barrier is the considerable complexity involved in data interpretation. Beyond these, the broader generalizability of research findings and the overall cost-effectiveness emerge as substantial impediments to its adoption as a routine clinical tool. Furthermore, enhanced diagnostic and assessment capabilities via artificial intelligence are transforming the evaluation of musculoskeletal health. Hip femoral neck fracture, a common osteoporotic fracture, is a critical area for assessing musculoskeletal health. While conventional CT provides structural bone information, BMD, and clear visualization of muscle tissue and fat infiltration, recent advancements in deep learning-based CT image analysis have significantly enhanced the quantitative assessment of proximal hip musculoskeletal tissues. These technologies, often employing deep learning architectures like convolutional neural networks (CNNs), are trained on large, diverse CT image datasets to achieve automated, high-precision segmentation and quantitative analysis of specific muscle groups (e.g., quadriceps, gluteal muscles, iliopsoas), bones, and different types of fat (e.g., intermuscular fat, subcutaneous fat). This method automatically measures key parameters. These include muscle cross-sectional area and muscle density. Muscle density, typically measured in Hounsfield units (HU), reflects the degree of fat infiltration. This approach allows for rapid and objective assessment of both muscle quantity and quality. Consequently, it aids in the early identification and evaluation of osteosarcopenia risk. This non-invasive, standardized quantitative assessment method holds promise for assisting in the development of more individualized prevention and treatment strategies (Pickhardt et al., 2020; Bridge et al., 2022; Imani et al., 2025). Despite this promising outlook, the generalizability of deep learning models across diverse imaging devices and patient cohorts requires rigorous validation to ensure their robustness. Concurrently, ethical considerations must be thoroughly addressed. Large-scale, multi-center clinical validation is paramount for securing regulatory approval and establishing the diagnostic efficacy of these techniques. Finally, regenerative medicine approaches are offering novel solutions for tissue repair. Addressing the challenge of smooth muscle defect repair, one study constructed a novel iron oxide nanowire/gelatin-silk fibroin composite hydrogel scaffold. Leveraging the geometric guidance of Fe_3_O_4_, combined with 3D bioprinting and magnetic induction, this scaffold enables the directed construction of biomimetic muscle tissue. The scaffold effectively promotes smooth muscle cell self-organization and the differentiation of bone marrow mesenchymal stem cells into smooth muscle cells, accelerating tissue regeneration and enhancing extracellular matrix deposition. This innovative strategy provides new avenues for the regenerative medicine treatment of osteosarcopenia (Luo et al., 2024). These advancements collectively represent a new frontier in understanding, diagnosing, and treating osteosarcopenia, moving towards more precise, personalized, and effective interventions. While regenerative medicine demonstrates immense potential in osteosarcopenia treatment, the long-term biocompatibility and immunogenicity of nanomaterials and cells require rigorous evaluation. A core challenge is whether biomimetic muscle tissue can achieve sufficient mechanical strength, neurovascularization, and physiological functional integration *in vivo*. Furthermore, standardized and high-cost large-scale production also represents a major impediment to its widespread application.

## 6 Conclusion

Osteosarcopenia represents the synchronized degeneration of bone and skeletal muscle. Its pathological progression is a process driven by a complex molecular interaction network within the bone–muscle unit. This review emphasizes that the pathogenesis of osteosarcopenia depends on multilevel bone–muscle crosstalk, encompassing autonomous intracellular regulation, local paracrine signaling at the bone–muscle interface, and systemic control by the neuroendocrine and metabolic axes. A comprehensive understanding of these interlinked pathways is essential for identifying precise therapeutic targets. Despite substantial advances in elucidating the molecular mechanisms underlying the bone–muscle unit, the field continues to face a profound translational gap between mechanistic discovery and clinical application. Current clinical management remains constrained by the global lack of a standardized diagnostic “gold standard,” and existing empiric interventions are insufficient to address the marked interindividual heterogeneity among patients. Furthermore, the absence of dual-action agents capable of simultaneously counteracting bone loss and muscle wasting significantly limits the overall therapeutic benefit. Future research should focus on employing spatial omics and multimodal imaging (e.g., deep learning–based CT analysis) to decode the bone–muscle crosstalk network, thereby advancing from mechanistic insight to subtype-specific diagnosis and individualized intervention. In parallel, the development of novel bioactive materials and targeted delivery systems with combined anabolic effects on both bone and muscle will be crucial for promoting synchronized regeneration within the bone–muscle unit—ultimately bridging the gap toward precision medicine in osteosarcopenia.
