# Supplementary material for: Osteosarcopenia: key molecular mechanisms and translational perspectives
Source: Front Physiol. 2026 Jan 9;16:1723522. doi: 10.3389/fphys.2025.1723522 (PMC12827509; doi:10.3389/fphys.2025.1723522)
Supplement: Supplementary file 1 [file Table1.docx]

Supplementary Material

# Supplementary Tables

## TABLE S1 Classification of Evidence Supporting Section 2: “Molecular Interaction Mechanisms”.

| Mechanistic Topic | Key Finding Summary | Evidence Type | Reference |
| --- | --- | --- | --- |
| Intracellular Autonomous Regulation: Spatiotemporal Activation and Signal Integration of the Wnt/β-Catenin Pathway | Wnt signaling induces β-catenin nuclear translocation and upregulates Runx2/Osterix expression | Review | (Hu et al., 2024) |
|  | Wnt activation suppresses osteoclastogenic RANKL signaling to preserve remodeling | Review | (Noh et al., 2020) |
|  | Age-related differences: reduced osteocytic Wnt activity, excessive Wnt in myogenic progenitors | Animal models | (Malvandi et al., 2025) |
|  | Targeted Wnt activation in BMSCs enhances osteogenic differentiation and bone repair | Animal models | (Matsushita et al., 2020) |
|  | Osteocytes secrete Wnt3a to activate β-catenin in neighboring cells | In vitro (Sukarawan); Review (Lin) | (Sukarawan et al., 2023;Lin et al., 2024) |
|  | SOST inhibits Wnt/β-catenin signaling, reducing bone repair; inhibition improves bone and muscle metabolism | Review | (Kirk et al., 2020;Sheng et al., 2023) |
| Bidirectional Regulation by Protein Mediators | Myokines from C2C12 myotubes activate osteocytic Wnt/β-catenin pathway | In vitro | (Lara-Castillo et al., 2023) |
|  | Increased muscle mass not always linked to improved performance (functional decoupling) | Human data | (Faraldi et al., 2025) |
|  | MSTN suppresses muscle growth (Smad2/3) and promotes osteoclastogenesis via RANKL | Review | (Saad, 2020;Cui et al., 2023) |
|  | MSTN KO mice show hypertrophy and normal contractility | Animal models | (Degens et al., 2025) |
|  | MSTN suppresses miR-218, increasing SOST/DKK1 and inhibiting Wnt signaling | Review | (Kirk et al., 2020) |
| miRNAs: A Novel Perspective in Bone-Muscle Metabolic Regulation | miR-214-3p downregulates CTNNB1 via LINC01133 axis | In vitro | (Guo et al., 2023;Chen et al., 2024;Tang et al., 2024) |
|  | miR-23a-5p inhibits osteogenic differentiation by targeting Runx2 | In vitro | (Yang et al., 2020) |
|  | Plasma miR-21, miR-23a, miR-24, miR-27a associated with bone density | Human data | (Nóbrega et al., 2020) |
| Inflammatory Factors: Regulating Bone-Muscle Balance | TNF-α, IL-1β, IL-6 promote osteoclastogenesis and muscle proteolysis | Review | (Jimenez-Gutierrez et al., 2022;Silva, 2024) |
|  | Chronic TNF-α reduces IL-15, promoting sarcopenic features | In vitro & animal models | (Alcalde-Estévez et al., 2025) |
| Systemic Regulation Layer | AgRP/NPY neuronal ablation increases bone mass | Animal models | (Enriquez et al., 2021) |
|  | Sympathetic signaling upregulates RANKL to link energy and bone metabolism | Review | (Shi and Chen, 2024) |
|  | Myokines and myometabolites modulate neuroinflammation and CNS energy homeostasis | Review | (Rai and Demontis, 2022) |
|  | Butyrate enhances bone/muscle anabolism via microbiota regulation | Animal models | (Chen et al., 2025) |
|  | Gut microbiota dysbiosis induces bone–muscle degeneration via inflammation | Review | (Li et al., 2024) |
|  | Anabolic bacterial species positively associated with lean mass and bone density | Human data | (Grahnemo et al., 2023) |

# SOST, sclerostin; BMSCs, bone marrow mesenchymal stem cells; RANKL, receptor activator of nuclear factor kappa B ligand; miR/miRNA, microRNA; DKK1, Dickkopf-related protein 1; CTNNB1, catenin beta 1; Runx2, runt-related transcription factor 2; BMD, bone mineral density; TNF-α, tumor necrosis factor alpha; IL-1β, interleukin-1 beta; IL-6, interleukin-6; NF-κB, nuclear factor kappa B; IL-15, interleukin-15; AgRP, agouti-related protein; NPY, neuropeptide Y; β2-AR, beta-2 adrenergic receptor; CNS, central nervous system; ALM, appendicular lean mass; SCFAs, short-chain fatty acids.

# References

Alcalde-Estévez, E., Moreno-Piedra, A., Asenjo-Bueno, A., Martos-Elvira, M., De La Serna-Soto, M., Ruiz-Ortega, M., et al. (2025). Aging-related hyperphosphatemia triggers the release of TNF-α from macrophages, promoting indicators of sarcopenia through the reduction of IL-15 expression in skeletal muscle. *Life Sci* 368**,** 123507.doi:10.1016/j.lfs.2025.123507

Chen, M., Li, Y., Zhai, Z., Wang, H., Lin, Y., Chang, F., et al. (2025). Bifidobacterium animalis subsp. lactis A6 ameliorates bone and muscle loss via modulating gut microbiota composition and enhancing butyrate production. *Bone Res* 13**,** 28.doi:10.1038/s41413-024-00381-1

Chen, S., Liu, H., Wang, Y., Wang, S., Yang, B., Sun, D., and Sun, P. (2024). Overexpression of lncRNA LINC00665 inhibits the proliferation and chondroblast differentiation of bone marrow mesenchymal stem cells by targeting miR-214-3p. *J Orthop Surg Res* 19**,** 2.doi:10.1186/s13018-023-04475-0

Cui, Y., Yi, Q., Sun, W., Huang, D., Zhang, H., Duan, L., et al. (2023). Molecular basis and therapeutic potential of myostatin on bone formation and metabolism in orthopedic disease. *Biofactors* 49**,** 21-31.doi:10.1002/biof.1675

Degens, H., Patel, K., and Matsakas, A. (2025). Myostatin Knockout Mice Have Larger Muscle Fibers With Normal Function and Morphology. *Muscle Nerve* 10.1002/mus.28389.doi:10.1002/mus.28389

Enriquez, R.F., Lee, N.J., and Herzog, H. (2021). AgRP signalling negatively regulates bone mass. *J Neuroendocrinol* 33**,** e12978.doi:10.1111/jne.12978

Faraldi, M., Provinciali, M., Di Rosa, M., Moresi, R., Sansoni, V., Gomarasca, M., et al. (2025). Circulating biomarkers associated with walking performance in elderly subjects: exploring miRNAs, metabolic and inflammatory biomarkers. *Geroscience* 47**,** 3977-3996.doi:10.1007/s11357-025-01510-2

Grahnemo, L., Nethander, M., Coward, E., Gabrielsen, M.E., Sree, S., Billod, J.M., et al. (2023). Identification of three bacterial species associated with increased appendicular lean mass: the HUNT study. *Nat Commun* 14**,** 2250.doi:10.1038/s41467-023-37978-9

Guo, Z., Li, J., Tan, J., Sun, S., Yan, Q., and Qin, H. (2023). Exosomal miR-214-3p from senescent osteoblasts accelerates endothelial cell senescence. *J Orthop Surg Res* 18**,** 391.doi:10.1186/s13018-023-03859-6

Hu, L., Chen, W., Qian, A., and Li, Y.P. (2024). Wnt/β-catenin signaling components and mechanisms in bone formation, homeostasis, and disease. *Bone Res* 12**,** 39.doi:10.1038/s41413-024-00342-8

Jimenez-Gutierrez, G.E., Martínez-Gómez, L.E., Martínez-Armenta, C., Pineda, C., Martínez-Nava, G.A., and Lopez-Reyes, A. (2022). Molecular Mechanisms of Inflammation in Sarcopenia: Diagnosis and Therapeutic Update. *Cells* 11.doi:10.3390/cells11152359

Kirk, B., Feehan, J., Lombardi, G., and Duque, G. (2020). Muscle, Bone, and Fat Crosstalk: the Biological Role of Myokines, Osteokines, and Adipokines. *Curr Osteoporos Rep* 18**,** 388-400.doi:10.1007/s11914-020-00599-y

Lara-Castillo, N., Masunaga, J., Brotto, L., Vallejo, J.A., Javid, K., Wacker, M.J., et al. (2023). Muscle secreted factors enhance activation of the PI3K/Akt and β-catenin pathways in murine osteocytes. *Bone* 174**,** 116833.doi:10.1016/j.bone.2023.116833

Li, Z., Wang, Q., Huang, X., Wu, Y., and Shan, D. (2024). Microbiome's role in musculoskeletal health through the gut-bone axis insights. *Gut Microbes* 16**,** 2410478.doi:10.1080/19490976.2024.2410478

Lin, W., Chow, S.K.H., Cui, C., Liu, C., Wang, Q., Chai, S., et al. (2024). Wnt/β-catenin signaling pathway as an important mediator in muscle and bone crosstalk: A systematic review. *J Orthop Translat* 47**,** 63-73.doi:10.1016/j.jot.2024.06.003

Malvandi, A.M., Vernillo, G., Sansoni, V., Faraldi, M., Verdelli, C., Coratella, G., et al. (2025). Metabolic Reprogramming for Body Adaptation and Inflammatory Control in Eccentric Damaging Exercise: Comprehensive Molecular Insights From Repeated Downhill Running. *MedComm (2020)* 6**,** e70480.doi:10.1002/mco2.70480

Matsushita, Y., Nagata, M., Kozloff, K.M., Welch, J.D., Mizuhashi, K., Tokavanich, N., et al. (2020). A Wnt-mediated transformation of the bone marrow stromal cell identity orchestrates skeletal regeneration. *Nat Commun* 11**,** 332.doi:10.1038/s41467-019-14029-w

Nóbrega, O.T., Morais-Junior, G.S., Viana, N.I., Reis, S.T., Perez, D.I.V., Freitas, W.M., et al. (2020). Circulating miR-34a and Bone Mineral Density of Brazilian Very-Old Adults. *J Aging Res* 2020**,** 3431828.doi:10.1155/2020/3431828

Noh, J.Y., Yang, Y., and Jung, H. (2020). Molecular Mechanisms and Emerging Therapeutics for Osteoporosis. *Int J Mol Sci* 21.doi:10.3390/ijms21207623

Rai, M., and Demontis, F. (2022). Muscle-to-Brain Signaling Via Myokines and Myometabolites. *Brain Plast* 8**,** 43-63.doi:10.3233/bpl-210133

Saad, F.A. (2020). Novel insights into the complex architecture of osteoporosis molecular genetics. *Ann N Y Acad Sci* 1462**,** 37-52.doi:10.1111/nyas.14231

Sheng, R., Cao, M., Song, M., Wang, M., Zhang, Y., Shi, L., et al. (2023). Muscle-bone crosstalk via endocrine signals and potential targets for osteosarcopenia-related fracture. *J Orthop Translat* 43**,** 36-46.doi:10.1016/j.jot.2023.09.007

Shi, H., and Chen, M. (2024). The brain-bone axis: unraveling the complex interplay between the central nervous system and skeletal metabolism. *Eur J Med Res* 29**,** 317.doi:10.1186/s40001-024-01918-0

Silva, R. (2024). The dichotomic role of cytokines in aging. *Biogerontology* 26**,** 17.doi:10.1007/s10522-024-10152-4

Sukarawan, W., Rattanawarawipa, P., Yaemkleebbua, K., Nowwarote, N., Pavasant, P., Limjeerajarus, C.N., and Osathanon, T. (2023). Wnt3a promotes odonto/osteogenic differentiation in vitro and tertiary dentin formation in a rat model. *Int Endod J* 56**,** 514-529.doi:10.1111/iej.13888

Tang, C., Huang, L., Guo, X.Q., Wang, G.G., and Chen, Z. (2024). LINC01133 promotes the osteogenic differentiation of bone marrow mesenchymal stem cells by upregulating CTNNB1 by acting as a sponge for miR-214-3p. *J Orthop Surg Res* 19**,** 572.doi:10.1186/s13018-024-05053-8

Yang, J.X., Xie, P., Li, Y.S., Wen, T., and Yang, X.C. (2020). Osteoclast-derived miR-23a-5p-containing exosomes inhibit osteogenic differentiation by regulating Runx2. *Cell Signal* 70**,** 109504.doi:10.1016/j.cellsig.2019.109504
